# Prevalence and risk factors for *Salmonella* spp. contamination in broiler chicken farms and slaughterhouses in the northeast of Algeria

**DOI:** 10.14202/vetworld.2018.1102-1108

**Published:** 2018-08-10

**Authors:** Samia Djeffal, Bakir Mamache, Rachid Elgroud, Sana Hireche, Omar Bouaziz

**Affiliations:** 1 GSPA Research Laboratory (Management of Animal Health and Productions), Institute of Veterinary Sciences, University Frères Mentouri Constantine 1, Constantine, Algeria; 2 Department of Veterinary Sciences, Institute of Veterinary and Agronomic Sciences, University Hadj Lakhdar, Batna, Algeria

**Keywords:** poultry, prevalence, risk factor, *Salmonella*, serotype, slaughterhouse

## Abstract

**Aim:**

The aim of this study was to provide information on the prevalence of *Salmonella* serotypes and to identify risk factors for *Salmonella* spp. contamination in broiler chicken farms and slaughterhouses in the northeast of Algeria.

**Materials and Methods:**

This study was conducted on 32 poultry farms and five slaughterhouses in the province of Skikda (northeastern Algeria). A questionnaire was answered by the poultry farmers and slaughterhouses’ managers. Biological samples (cloacal swabs, droppings, caeca, livers, and neck skins) and environmental ones (water, feed, surface wipes, rinsing water, and sticking knife swabbing) were taken to assess the *Salmonella* contamination status.

**Results:**

Nearly 34.37% of the poultry farms and all the slaughterhouses were contaminated with *Salmonella*. The isolated *Salmonella* strains belonged to two major serotypes: Kentucky and Heidelberg followed by Enteritidis, Virginia, and Newport. There was an evident heterogeneous distribution of serotypes in poultry farms and slaughterhouses. Only one factor (earth floor) was significantly associated with *Salmonella* contamination in poultry houses (p<0.05).

**Conclusion:**

A high prevalence rate of *Salmonella* contamination was found in poultry farms and slaughterhouses in Skikda region. These results showed the foremost hazardous role of poultry production in the spread and persistence of *Salmonella* contamination in the studied region.

## Introduction

*Salmonella* spp. is important foodborne pathogens distributed worldwide that frequently infect poultry flocks. Epidemiological studies have demonstrated several routes through which *Salmonella* spp. can be disseminated within poultry flocks [1]. Birds become infected by horizontal transmission through infected litter, feces, feed, water, dust, fluff insects, equipment, fomites, diseased chicks, and *Salmonella* contaminated rodents [2]. Other domestic animals, wild birds, and personnel can transmit *Salmonella* spp. to broiler chicken throughout the rearing period. Vertical transmission from parent flocks results mostly from ovarian transmission or through the eggshell after laying [3].

It is essential to take into account the problem of contamination of livestock both for its impact on public health and for its significant economic repercussions [4]. However, if contamination of meat is possible at all levels of the production chain, the rearing periods represent the main critical steps for the contamination burden by *Salmonella* [5]. The knowledge of the modalities of contamination of the broiler chicken by this pathogen during each period of the production is essential to prevent infection.

This study provides additional epidemiological information on *Salmonella* contamination of broiler flocks and slaughterhouses in Skikda region and gives a complete serological distribution of this pathogen which has been previously revealed by several studies in other regions of the country (Constantine, Annaba, and Batna) [6-8]. Compared to these previous studies, the present survey investigated the prevalence of *Salmonella* spp. in broiler chicken farms and slaughterhouses during two different age periods of the broiler chicken used a different sampling methodology and more exhaustive sites of sampling. In addition, this study has demonstrated that broiler flocks and slaughterhouses of the region of Skikda were infected with different serovars. The antibiotic resistance profiles of Skikda’s *Salmonella* isolates showed a marked difference with those of other Algerian provinces [9].

Since 1980, the Algerian poultry industry has experienced a remarkable development supported by a governmental encouragement policy. These dynamics have resulted in a sharp increase in the poultry industry and production with a remarkable shift from farm type and family poultry farming to intensive poultry farming to ensure low-cost animal proteins to the consumer. Chicken meat is more popular than red meat because of its relatively lower price and easier digestibility. It is worth noting that this technological development is limited only to the state sector. However, private flock breeding and slaughtering systems are still suffering from substantial technological backwardness which profoundly affects the productivity of this sector and reduces the hygienic quality of the poultry products with massive consequences on the public health. In fact, the hygienic quality of the poultry products depends on the breeding conditions and management, and particularly on the conception and the hygienic status of the poultry houses [10]. The region of Skikda is characterized by its specific climate with very humid and mild winters and dry and very hot summers. These environmental conditions affect the microbial persistence in the broiler houses and affect the chicken bacterial colonization and health.

The aim of the present study was to estimate the prevalence of *Salmonella* spp. infection in broiler chicken farms and slaughterhouses and to identify the risk factors among farm characteristics and management practices that are associated with this infection in Skikda region. For this reason, questionnaires were established on closed-type questions basis related to all breeding and slaughtering conditions. They were addressed to all broiler chicken farmers and slaughterhouses’ managers.

## Materials and Methods

### Ethical approval

This study was conducted according to ethical guidelines that were controlled and approved scientific council of the Institute of Veterinary Sciences (Mentouri Brothers University, Constantine - 1, Algeria) and complied with the guidelines for animal care and use in research and teaching. It is worth noting that no live birds were used in this study.

### Study area

This survey was carried out from December 2011 to May 2013. The target population included 32 broiler chicken farms and five chicken slaughterhouses in the province of Skikda (northeastern Algeria). We have tried to cover all the localities and the majority of poultry farms and slaughterhouses. The choice of poultry farms and slaughterhouses was guided by the free manager’s acceptation to cooperate or not to the study.

### Data collection and sampling conditions

Each farm was visited twice for sampling at two age periods (15-30 days and 45-60 days). The slaughterhouses were visited only once, and the managers were interviewed with the questionnaire at the time of sampling.

The epidemiological questionnaire for broiler farms contained 60 closed-type questions. The latter was related to the location and conception of the rearing buildings, the broiler rearing characteristics: Equipment, environmental conditions, biosecurity measures, origin of chicks and feed, farm staff, vaccination programs, and use of antibiotics. For the slaughterhouses, another questionnaire was filled out. It concerned the design of the infrastructure, construction materials, equipment, slaughter conditions, cleaning and disinfection methods, carcass inspection, and personnel qualifications.

The information thus gathered was based both on personal observations and on data collected from breeders and veterinarians who provide medical monitoring of livestock and sanitary inspection of the slaughterhouses.

A total of 1194 and 90 samples were collected from the poultry houses and the slaughterhouses, respectively. The sampling procedures were standardized, and the samples were taken randomly. The matrices differed and were constituted mainly by droppings (1 pool of 5 g×5), cloacal swabs (1 pool of swab×5), surface rags (1 pool of wipes 25 cm×25 cm, AES Chemunex, Combourg, France), water from drinking troughs (1 pool 25 mL×5), food from feeders (1 pool of 5 g×5), caeca, liver, neck skin (1 pool of 5 g×5), and sticking knife swabs (1 pool). All samples were analyzed in the same bacteriology laboratory with the same analytical methods.

All samples were transported to the laboratory, on ice packs within a period not exceeding 2 hours to be treated on the same day or kept in the refrigerator overnight. The organization of sampling in farms and poultry slaughterhouses is shown in Table-1.

**Table-1:** Organization of sampling in farms and poultry slaughterhouses.

Nature of building	Number of building	Nature of samples	Number of samples
Poultry houses	32	Cloacal swabs	330
		Food	160
		Water	320
		Wipes	64
		Feces	320
Total	32	Total	1194
Slaughterhouses	5	Caecum	25
		Liver	25
		Neck skin	25
		Carcass rinse water	5
		Sticking knife swabs	5
		Wipes swabbing	5
Total	5	Total	90

### Salmonella isolation and identification

Bacteriological analyses were performed according to the protocols set by the International Organization for Standardization [11] for *Salmonella* detection in food and animal feedstuffs. 25 g of samples (droppings, feed, liver, caeca, and neck skin) were separately pre-enriched with 225 mL of buffered peptone water broth (PWB) (Fluka, Sigma-Aldrich, France). The swabs were placed individually in 10 mL PWB, while 100 mL of drinking and carcass rinsing waters were individually mixed with 100 mL of double strength PWB for pre-enrichment according to NF U 47-101 Standard [12]. All the samples were incubated at 37°C for 18-20 h. From each pre-enrichment solution, 1 mL and 0.1 mL were, respectively, transferred into 10 mL of enrichment Muller-Kauffmann Tetrathionate/Novobiocin broth (AES Chemunex, Combourg, France) and 10 mL of Rappaport-Vassiliadis broth (Merck Darmstadt, Germany) and incubated at 37°C and 42°C for 24 h, respectively. Both enriched samples were then streaked on XLD (Fluka analytical Steinheim, Switzerland) and Hektoen agars (Pasteur Institute of Algeria) and incubated at 37°C for 24 h. *Salmonella -* suspected colonies were identified with the API 20E System (bioMérieux, France). Confirmed *Salmonella* isolates were serotyped according to the Kauffmann-White-Le Minor’s scheme [13].

### Statistical analysis

Differences in contamination levels of poultry houses at the two sampling periods (15-30 days vs. 45-60 days), and risk factors for *Salmonella* spp. contamination were assessed by the Chi-square test (at 95% CI and p<0.05). All statistical analyses were performed using the EpiInfo™ software (2016).

## Results

### Characteristics of the poultry farms and slaughterhouses

Twenty-seven poultry houses have concrete walls and floors, and corrugated metal sheet roofs and five ones have clay floors with walls and roofs made of straw and reed covered with plastic foil. Their rearing capacity varies from 3500 to 20,000 birds per house. Most of them produced four grow-out houses per year. In 22 of 32 broiler houses, stocking density was more than 10 birds/m^2^. Poultry farms consisted of 1-2 houses without fence allowing other domestic (cattle, sheep, dogs, and cats) and wild animals to have contact with birds. The poultry farm belonging to the public sector consisted of 4 fenced houses.

Strict biosecurity measures were applied in studied farms. However, we noticed the absence of disinfection foot dip except in two poultry houses. All slaughterhouses have concrete floors with earthenware walls; others have tiled walls except for two that had cement floor and walls, which are difficult to clean. They are equipped with a mechanized felling chain and a cold room. The slaughtering capacity ranges from 2000 to 7000 chickens per day, and some of the broilers are brought from different poultry farms of the neighboring provinces.

### Prevalence of Salmonella serotypes in broilers, slaughterhouses, and human samples

Eleven (34.37%) of the 32 poultry farms were contaminated with *Salmonella*. The isolation rate varied according to the origin of the sample: 6.25% for wipes (n=64), 3.93% for swabs (n=330), 3.12% for droppings (n=320), and 2.18% for water samples (n=320) and all the feed samples were free of *Salmonella* (n=160). The samples taken at the age of 3 weeks were more contaminated than those collected at the end of the raising period. *Salmonella* spp. was isolated in all the slaughterhouses with contamination of different matrices: Caeca (12%, n=25), neck skin (8%, n=25), livers (4%, n=25), wipes (40%, n=5), sticking knives (20%, n=5), and carcass rinsing water (20%, n=5).

**Figure-1:**
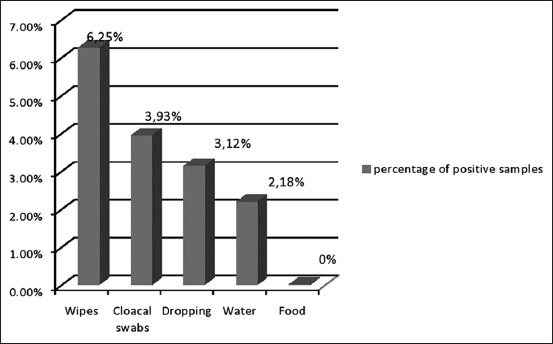
Percentage of positive samples collected from poultry farms.

**Figure-2:**
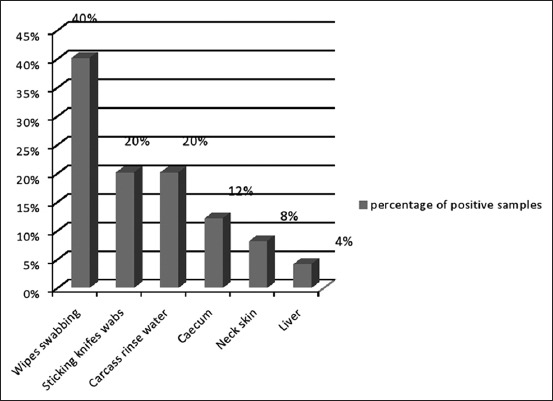
Percentage of positive samples collected from slaughterhouses.

The percentage of positive samples collected from poultry farms and slaughterhouses is shown in Figures-1 and 2. 45 *Salmonella* strains were isolated from broiler chicken farms and slaughterhouses. Most of them were *Salmonella* Kentucky (n=22) and *Salmonella* Heidelberg (n=13), and the remaining strains were of *Salmonella* Virginia (n=5), *Salmonella* Enteritidis (SE) (n=4), and *Salmonella* Newport (n=1). There was a heterogeneous distribution of some serotypes between poultry farms and slaughterhouses.

### Univariate analysis of the factors favoring Salmonella contamination

The results of the univariate analysis of the association between the explanatory variables and the presence/absence of *Salmonella* are summarized in Table-2. From the questionnaires, 10 potential risk factors for livestock and slaughterhouses were identified. After calculating the odds ratios (ORs) of the assumed risk factors, we were able to determine six risk factors (age, water use from tanks and wells, number of bands per year, density, chicken house structure, and soil type).

**Table-2:** Risk factors for *Salmonella* contamination.

Risk factors	Positive poultry houses	Negative poultry houses	Chisquare	Pα	OR	Confidence interval
Age						
15-30 days	10	22	0.30	0.05	1.36	[0.44-9.55]
45-60 days	8	24				
Water source						
Tank water	8	14	0.11	0.05	1.33	[0.24-7.21]
Tap water	3	7				
Number of bands year						
1/year	1	1	0.23	0.05	2	[0.11-36]
2 or more/years	10	20				
Density						
810 birds/m^2^	8	18	0.79	0.05	2.25	[0.37-13.61]
>10 birds/m^2^	3	3				
House structure						
Hoop house	3	2	1.74	0.05	1.78	[0.58-4.20]
Cement block	8	19				
Soil type						
Clay floors	5	5	4.40	0.05	2.66	[1.07-6.60]
Concrete	6	16				

All the factors studied appeared to be relevant and tended to be potentially associated with contamination of farms by *Salmonella*. However, only one indicator (clay floor) was found to be statistically significant, the other factors associated with contamination were not significant. This could be due to recruitment bias during the epidemiological investigation (incomplete questionnaires and in interpreting of the questions).

## Discussion

This study has provided epidemiological data and identified risk factors favoring the infection of broiler farms by *Salmonella* spp. in Skikda region. This cross-investigation suggested some prophylactic measures to reduce contamination of the broiler farms. The number of broiler farms was high in this region. Due to some economic constraints, we limited ourselves to covering some municipalities whose farms were readily accessible and whose owners tolerated voluntary participation in this study. It is worth noting that no farmers were aware of the flock’s contamination status. We guaranteed the anonymity and the absence of any negative repercussions of the results obtained on their activity. Although the number of surveyed flocks conformed to the target, the number of farmers studied was definitely lower. This low number of flocks involved a loss of statistical power and may explain the absence of association between the exposure to certain factors and flocks’ contamination. The use of questionnaires for the collection of baseline data could also introduce bias; even so, most questions were objective and close.

The estimated prevalence of *Salmonella* spp. positive chicken flocks and slaughterhouses was 34.37% and 100%, respectively. Further investigations that could overcome such stated deficiencies would be necessary to solidly reflect the epidemiological risks of *Salmonella* in this area [6, 7, 14, and 15]. The *Salmonella* spp. prevalence varied considerably among regions and countries such as in several European countries. The reported prevalence was in France (3.4%), Italy (9.2%), Germany (2.7%), Spain (1.02%), and Sweden (nearly 0%) [16, 17].

Regarding the results of the study of the questionnaires, this prevalence could be explained by the low hygienic level of the studied poultry houses, their structures, especially that some of them are hoop houses, non-regulated livestock buildings (OR 1.78), and by the absence of sanitary control measures. Chickens can be contaminated horizontally during the rearing period through litter, food, drinking water, dust, and contaminated equipment [2].

The occurrence of the various *Salmonella* serotypes in the studied poultry farms and slaughterhouses was *Salmonella* Kentucky (49%), *Salmonella* Heidelberg (29%), *Salmonella* Virginia (11%), SE (9%), and *Salmonella* Newport (2%). The heterogeneity in *Salmonella* distribution between poultry farms and slaughterhouses suggested that most of *Salmonella* serotypes were transmitted before the slaughter process. *Salmonella* Kentucky was the most predominant. This serotype has been associated with the chicken industry in the USA [18], but now, its distribution is worldwide (Africa, the Middle East, and Europe) especially its ST198 [19].

In this study, *Salmonella* Heidelberg has been isolated only in poultry farm. This serotype has been isolated in previous studies in some provinces of the center of Algeria [20]. Although we have isolated SE in only 9% of the samples, Putturu *et al*. [21] reported a higher incidence of SE in chicken feces and cloacal samples of poultry in Hyderabad (India). It is worth noting that it is the most common serotype in animal products especially in poultry [16]. During the mid-1980s, this serotype has emerged as a result of vertical and horizontal transmissions within and between large poultry flocks in many parts of the world [22, 23].

*Salmonella* Newport was isolated in one sample of the poultry farms studied. This serotype has rapidly emerged as a pathogen in both animals and humans throughout the United States [24]. Six risk factors for *Salmonella* contamination of the poultry flocks were identified, but only the nature of soil (clay floor) was statistically significant. In a study conducted by Huneau-Salaủn *et al*. [25], 51.7% of the positive flocks reared on on-floor farms with a history of SE contamination were again found to be due to SE. *Salmonella* might persist in contaminated poultry houses where the standards of cleaning and disinfection are unsatisfactory [26]. The persistence of *Salmonella*, especially in the open-air range, might be a source of contamination of flocks in on-floor farms. Although the chicken is free of *Salmonella*, infection is possible with a poorly cleaned and poorly disinfected building, despite the crawlspace, with the bacteria surviving more than 6 months in an outdoor environment [27]. Alternative or synergistic effects may be due to the role of soil in determining the risk of *Salmonella* introduction into broiler houses on mechanical vehicles or with living reservoirs. For the former, characteristics of the soil at broiler farm location may impact on the risk of *Salmonella* being brought into the houses on such mechanical vehicles as farm worker footwear or movable equipment [28]. During our investigation, we noted that the hygiene measures were applied in the bare minimum and the absence of footbath except in two poultry houses.

The samples taken at the age of 15-30 days were more contaminated than those collected at 45-60, days but the difference was not significant (p>0.05). *Salmonella* colonize easily the entire digestive tract and can be isolated more easily; especially as they are excreted intermittently in fresh droppings [29]. It should also be noted that the droppings were harvested from the poultry houses with clay floors. This represents a statistically significant risk factor (OR=2.66), which is in accordance with the results reported by Elgroud *et al*. [6], (OR=21) in a study conducted on broiler houses in Constantine region.

In a study in Morocco [14] reported that the contamination of the previous breeding band seems to significantly increase the risk of contamination by *Salmonella* spp. (OR=5.14), and contaminated chicks represented also an important risk factor (OR=10.5). They contribute to the increase in the level of contamination of livestock buildings through their manure [30-32]. In this study, we found *Salmonella* in the feces samples, confirming the importance of the horizontal contamination in a breeding band [26, 29].

Environmental sampling has been reported to be a good indicator of the presence of *Salmonella* in poultry flocks [33]. Andres and Davies (2015) [34] reported that *Salmonella* was ubiquitous in a farm environment. The density of chickens, especially when it is <0 chickens per square meter is also an important risk factor (OR=2.25). Although in our study, this factor was not statistically significant (p>0.05) to the recruitment bias of our farms, Elgroud *et al*. [6] noted that it was significant (OR=7.7). This finding corroborated with the study of Chaiba and Rhazi-Filali [14] (OR=7.7), mentioning that the poultry houses whose density is below 25 subjects per square meter have a lower contamination rate than that of farms with densities >25 m^2^, which is in agreement with the literature relating that high density in a chicken house is a factor favoring contamination by *Salmonella* [35].

The drinking water derived, for the majority of the farms studied from sources, wells, and cisterns, which were monitored neither by farmers nor by the competent sanitary services. Chaiba and Rhazi-Filali [14] noted that poultry farms using network water from the national drinking water agency had a lower prevalence of *Salmonella*. Argüello *et al*. [36] reported that the treatment of water mainly by the addition of organic acids reduced the number of *Salmonella*. No *Salmonella* was isolated in samples of animal feed. In Batna Province (northeastern Algeria) and France, this is in agreement with a study conducted in Batna Province (northeastern Algeria) [7] and France [25].

It should be noted that some risk factors for contamination of a flock are difficult to prove statistically because of the scarcity of samples. Nevertheless, they may be important in particular cases such as the presence of rodents in the broiler house of one flock [32]. *Salmonella* transmission and contamination can be enhanced by the situation of poultry houses. Practices such as overcrowding, unhygienic farming activities, lack of adequate biosecurity measures, and equipment worsen the situation. Mice, wild birds, ants, and snakes have been demonstrated in some studies, to be important agents for transmission of *Salmonella* among avian flocks [37-39]. On the other hand, environmental samples such as droppings, soil, crevices, dust, litters, feeders, and/or drinkers may harbor *Salmonella* and increase the contamination rate [40].

For the slaughterhouses, we could not adopt statistical analyses since all of them were contaminated, and all the factors could have been involved in the salmonella contamination. This finding corroborated the results of Elgroud *et al*. [6], who reported a prevalence of *Salmonella* contamination of 73.33% in poultry slaughterhouses in Constantine region.

According to many surveys, contamination of poultry products with *Salmonella* may take place at different stages of the production period [41]. After contamination of birds at the farm, bacteria colonize the intestine and can contaminate the carcasses during slaughtering. Cross-contamination is also possible [42].

## Conclusion

This study provides important epidemiological information on the *Salmonella* contamination status in the broiler chicken farms and slaughterhouses of the region of Skikda. Serotyping provides a better understanding of the epidemiology of the disease, and by measuring the trends of serotypes over time, information about emerging serotypes, and the efficacy of control programs can be obtained. Salmonellosis is a management disease, and its control depends on controlling the sources of contamination and transmission. Factors related to herd contamination are generally linked to biosecurity measures and the design of the premises where they are controlled, thus preventing the introduction, survival, and multiplication of germs or their vectors in livestock and slaughterhouses. It is fundamental to respect good hygiene practices and to apply sanitary regulations upstream of the broiler chicken industry to provide hygienic meat and meat products to the consumer.

## Authors’ Contributions

SD has actively worked on the isolation of avian *Salmonella* strains, identification of strains, data interpretation, drafting the paper, and revising it. BM conceived and designed the study and revised the paper. SH participated actively in data interpretation and contributed in part to the draft writing. RE and OB conceived and designed the study. All authors read and approved the final manuscript.
